# *Ca*. Similichlamydia in Epitheliocystis Co-infection of Gilthead Seabream Gills: Unique Morphological Features of a Deep Branching Chlamydial Family

**DOI:** 10.3389/fmicb.2017.00508

**Published:** 2017-03-30

**Authors:** Helena M. B. Seth-Smith, Pantelis Katharios, Nancy Dourala, José M. Mateos, Alexander G. J. Fehr, Lisbeth Nufer, Maja Ruetten, Maricruz Guevara Soto, Lloyd Vaughan

**Affiliations:** ^1^Vetsuisse Faculty, Institute for Veterinary Pathology, University of ZürichZürich, Switzerland; ^2^Functional Genomics Center Zürich, University of ZürichZürich, Switzerland; ^3^Hellenic Center for Marine Research, Institute of Marine Biology, Biotechnology and AquacultureHeraklion, Greece; ^4^Selonda AquacultureAthens, Greece; ^5^Center for Microscopy and Image Analysis, University of ZürichZürich, Switzerland; ^6^Pathovet AGTagelswangen, Switzerland; ^7^Department of Infectious Diseases and Pathobiology, Centre of Fish and Wildlife Health, University of BernBern, Switzerland

**Keywords:** epitheliocystis, *Planctomycetes-Verrucomicrobia-Chlamydiae*-superphylum, Piscichlamydia, Similichlamydia

## Abstract

The *Planctomycetes-Verrucomicrobia-Chlamydiae* (PVC) bacterial superphylum constitutes a broad range of organisms with an intriguing array of ultrastructural morphologies, including intracellular membranes and compartments and their corresponding complex genomes encoding these forms. The phylum *Chlamydiae* are all obligate intracellular bacteria and, although much is already known of their genomes from various families and how these regulate the various morphological forms, we know remarkably little about what is likely the deepest rooting clade of this phylum, which has only been found to contain pathogens of marine and fresh water vertebrates. The disease they are associated with is called epitheliocystis; however, analyses of the causative agents is hindered by an inability to cultivate them for refined *in vitro* experimentation. For this reason, we have developed tools to analyse both the genomes and the ultrastructures of bacteria causing this disease, directly from infected tissues. Here we present structural data for a member of the family *Ca*. Similichlamydiaceae from this deep-rooted clade, which we have identified using molecular tools, in epitheliocystis lesions of gilthead seabream (*Sparus aurata*) in Greece. We present evidence that the chlamydial inclusions appear to develop in a perinuclear location, similar to other members of the phylum and that a chlamydial developmental cycle is present, with chlamydial forms similar to reticular bodies (RBs) and elementary bodies (EBs) detected. Division of the RBs appeared to follow a budding process, and larger RBs with multiple condensed nucleoids were detected using both transmission electron microscopy (TEM) and by focused-ion beam, scanning electron microscopy (FIB-SEM). As model hosts, fish offer many advantages for investigation, and we hope by these efforts to encourage others to explore the biology of fish pathogens from the PVC.

## Introduction

Epitheliocystis is a common disease of wild and cultured fish, characterized by cyst-like inclusions in gill and skin epithelium. It was first described in bluegill (*Lepomis machrochirus*) (Hoffman et al., 1969) and since then has been observed in more than 80 different fish species (Stride et al., 2014) in both marine and freshwater environments (Nowak and LaPatra, 2006). The first studies combined molecular and morphological techniques to provide firm supportive data for *Chlamydia*-like organisms, reflected in the naming of *Candidatus* Piscichlamydia salmonis (Draghi et al., 2004) and *Candidatus* Clavichlamydia salmonicola (Karlsen et al., 2008). At the same time, these two new species also defined new families which could not better illustrate the diversity amongst a rapidly expanding phylum *Chlamydiae*.

The *Ca*. Clavichlamydiaceae remain the closest water-based relatives and immediate ancestors to the *Chlamydiaceae*, chlamydial pathogens of land vertebrates and major human disease agents (Taylor-Brown et al., 2015). This is also reflected in their morphologies, with predicted chlamydial developmental cycles, including peripheral, dividing reticular bodies (RBs), intermediate bodies (IBs), and the infectious particles or elementary bodies (EBs), the latter showing the head and tail form only observed in *Ca*. Clavichlamydia salmonicola until now (Karlsen et al., 2008; Schmidt-Posthaus et al., 2012; Guevara Soto et al., 2016).

The *Ca*. Piscichlamydiaceae, in contrast, were the first representatives of a new deeply branching clade, found exclusively in marine and fresh water vertebrates. A chlamydial developmental cycle was evident, although this differed in key aspects to the *Ca*. Clavichlamydiaceae, including cytoplasmic bridges linking dividing or budding RBs found throughout the inclusion and a differing host response (Schmidt-Posthaus et al., 2012), indicating that this group of bacteria utilizes a different pathological armory. An exciting development was the recent addition of new candidate families to this deep-branching clade, variously found in Southern hemisphere waters and including the *Ca*. Parilichlamydiaceae infecting yellow tail kingfish (*Seriola lalandi*) (Stride et al., 2013a), members of the *Ca*. Similichlamydiaceae infecting striped trumpeter (*Latris lineata*) and barramundi (*Lates calcarifer*) (Stride et al., 2013b,c), *Ca*. Actinochlamydiaceae causing epitheliocystis in catfish (*Clarius gariepinus*) in a Ugandan lake system (Steigen et al., 2013), and new species of the *Ca*. Similichlamydiaceae infecting ballan wrasse (*Labrus bergylta*) used as cleaner fish in salmon farms off the Norwegian coast (Steigen et al., 2015). These all share the property of stimulating a moderate host response, although the nature of the chlamydial inclusions and their developmental cycles differ from other *Chlamydia*.

Early studies describing the diverse cyst types and bacterial forms detected in sparid (Paperna, 1977; Crespo et al., 1999) and salmonid (Nylund et al., 1998) epitheliocystis assumed the causative organisms primarily to belong to the phylum *Chlamydiae*, but also raised the possibility of mixed infections with rickettsia-like bacteria containing cysts. The careful study of mixed epitheliocystis infections in Norwegian salmon (Steinum et al., 2010), pointing to an as yet unknown agent, led to the first identification of a beta-proteobacterium causing epitheliocystis (Toenshoff et al., 2012). Other species of beta-proteobacteria have since been identified in mixed epitheliocystis infections of gilthead seabream (*Sparus aurata*) (Qi et al., 2016; Seth-Smith et al., 2016), as have gamma-proteobacteria been identified in cysts from lake trout (*Salvelinus namaycush*) (Contador et al., 2016), Cobia larvae (Mendoza et al., 2013), and sharpsnout seabream (*Diplodus puntazzo*) larvae (Katharios et al., 2015). In none of these bacterial infections were chlamydial developmental cycles evident.

Epitheliocystis in gilthead seabream, which is one of the major cultured species in the Mediterranean, was first reported several decades ago (Paperna, 1977; Crespo et al., 1999). During a recent survey of the disease in cultured gilthead seabream in Greece during 2012–2013 we identified a more complex disease scenario with several bacterial species co-infecting the fish cohort gills. Using 16S rRNA gene sequencing combined with fluorescent *in situ* hybridization (FISH), we identified two species of a novel beta-proteobacterial genus, *Candidatus* Ichthyocystis, as the main pathogens (Qi et al., 2016; Seth-Smith et al., 2016). Complicating the analysis even further, new species of the *Ca*. Piscichlamydiaceae and *Ca*. Similichlamydiaceae were also identified molecularly, of which the latter was the dominant chlamydial agent and these results we now present here. FISH with a probe recognizing both families localized these agents to morphologically separable inclusions from the dominant *Ca*. Ichthyocystis agents (Seth-Smith et al., 2016, Supplementary Figure 3). We now present detailed molecular, transmission electron microscopy (TEM) and focused ion beam-scanning electron microscopy (FIB-SEM) analyses to reaffirm the unique aspects of the *Ca*. Similichlamydia developmental cycles which, although clearly chlamydial in nature, share features found in representatives of the *Planctomycetes*, raising questions concerning the mechanisms of cell division in the *Chlamydiae*. Although epitheliocystis has been reported in Greece and in gilthead seabream, this is the first report with molecular evidence connecting the disease with members of the *Chlamydiae*.

## Methods

### Fish sampling

Sampling of juvenile gilthead seabream (*S. aurata*) was carried out in Selonda SA farms in Saronikos (sampled November 2012 randomly; 2012Sar) and Argolida (sampled June 2013 during an epitheliocystis outbreak; 2013Arg), as previously described (Seth-Smith et al., 2016). Sampling formed part of the routine monitoring of fish health on the farms by one of the authors (ND). Gill arches from individual fish were taken in parallel into 10% buffered formalin, RNALater and pure ethanol, with the 2013Arg samples also taken into M4RT *Chlamydia* transport medium (Remel Microtest M4RT, Thermo Fisher, Switzerland) and sterile sea water and sent chilled by overnight courier to Switzerland. All samples were kept chilled and further processed immediately upon arrival.

### TEM and histology

Screening of gill samples with standard histology was performed on samples fixed in 10% neutral buffered formalin, followed by dehydration in an ascending alcohol series ending in xylol and afterwards embedded in paraffin. Paraffin blocks were cut in 2–3 μm thin sections, mounted on glass slides and stained using a routine protocol for haematoxylin and eosin (HE) staining.

For electron microscopy, formalin-fixed gill tissues were post-fixed in a mixed solution of 1% paraformaldehyde and 2.5% glutaraldehyde in 0.1 M sodium phosphate buffer, pH 7.5 at 4°C overnight. Samples were prepared for TEM by embedding into epoxy resin according to standard procedures (Seth-Smith et al., 2016). Epoxy resin blocks were screened for epitheliocystis lesions using semithin sections (1 μm) which were stained with toluidine blue (Sigma-Aldrich). Ultrathin sections (80 nm) were mounted on copper grids (Merck Eurolab AG, Dietlikon, Switzerland), contrasted with uranyl acetate dihydrate (Sigma-Aldrich), and lead citrate (Merck Eurolab AG) and investigated using a Philips CM10 transmission electron microscope. Images were processed with Imaris 7.6.1 (Bitplane, Oxford Instruments) and assembled into panels for publication and annotated using Photoshop (Adobe).

### Focused ion beam-scanning electron microscopy (FIB-SEM)

Sample blocks were fixed in 2.5% glutaraldehyde in 0.2 M sodium cacodylate buffer, pH 7.4 at room temperature overnight, followed by 1% osmium tetroxide, and contrasted with 2% aqueous uranyl acetate. Sample dehydration was in an ethanol series, then propylene oxide and embedded in Epon 812 resin. Semithin and ultrathin sections were obtained to identify the regions containing cysts. The selected blocks were attached to 12 mm stubs by conductive carbon cement followed by carbon coating. 3D datasets were acquired with a FIB-SEM Auriga 40 Crossbeam (Zeiss, Oberkochen, Germany) using the FIBICS Nanopatterning engine (Fibics Inc, Ottawa, Canada). The gallium-ion beam for milling was used at 30 kV, 600 pA current and the images were acquired at an acceleration voltage of 1.5 kV using an in-lens energy selective backscattered electron detector (ESB) with a grid voltage of 1.3 kV. The resolution was set to 5 nm in the XY axes and 5–10 nm in the Z axis. The image stacks were aligned with TrackEM2 (Cardona et al., 2012). The aligned dataset was visualized with Imaris 7.6.1. To better visualize the shape of the bacteria as well as for showing a dividing bacterium the FIB-SEM dataset was segmented with the software Ilastik, 1.1 (Sommer et al., 2011).

### Bacterial identification

Identification of chlamydial bacteria was carried out using *Chlamydiae*-specific 16S rRNA gene primers (Everett et al., 1999; Draghi et al., 2004). Positive bands were analyzed further only when template free negative controls showed no signal. 16S rRNA gene amplicons were cloned into Topo vector pCR2.1 prior to capillary sequencing from both ends (Microsynth, Balgach). The resulting reads were assembled (CLC Main Workbench 7.0.2, CLC bio, Qiagen), compared using blastn against the Genbank database and used to create alignments with reference sequences using Muscle and PhyML v3 within Seaview v4 (Gouy et al., 2010). Only unique sequences from each sample were included in the analysis. Representative 16S rRNA gene sequences have been deposited with EMBL with the following accession numbers: LN612731-4 representing 2012Sar3_5c, 2013Arg23_1c, 2013Arg33_2c, and 2013Arg14_4c respectively.

### Fluorescent *in situ* hybridization (FISH)

FISH was performed on the Ventana Discovery XT automated platform using the probe shown in Table 1. Automated deparaffinization was followed by pretreatment with 0.2 M HCl in PBS then pepsin (500 μg ml ^−1^ in this buffer) for 4 min. Following addition of 50 ng probe per slide in a hybridization buffer comprising 6 × SSC, 5 × Denhardt's solution, and 12% dextran sulfate, samples were denatured at 90°C for 4 min and hybridized at 48°C overnight. A wash with 2 × SSC at 48°C was followed by manual post staining with 4′-6-diamin-2-phenylindole (DAPI) at 10 μgml^−1^ to visualize bacterial and host DNA for 10–30 min.

**Table 1:** **Probe used in FISH**.

**Probe**	**Specificity (sp)**	**Sequence/fluorophore**	**Position (*E.coli* numbering)**	**Reference**
Pisci-0312	*Ca*. Piscichlamydia/Similichlamydia	5′-AGTCCCAGTGTTGGCGATCG-3′Cy3	304–323	Seth-Smith et al., 2016

FISH and HE sections were scanned on a Hamamatsu Nanozoomer 2.0 HT scanner for an overview of the section, and high resolution imaging was performed on the Leica SP5 resonant confocal laser scanning microscope. Deconvolution was performed using Huygens (Ponti et al., 2007) and images were prepared with Imaris 7.6.1 (Bitplane, Oxford Instruments) and assembled into panels for publication and annotated using Photoshop CS4 extended, version 11.0.2 or CS6 extended, version 13.0x32 (Adobe).

## Results

### Identification of chlamydial agents present in infected gills

In total, five gilthead seabream gill samples from Saronikos bay (2012) and 16 samples from Argolida (2013) were screened for the presence of chlamydial sequences. All samples had visible epitheliocystis lesions under histopathological examination, although infection intensities were higher in Argolida fish. The majority of the cysts were attributed to *Candidatus* Ichthyocystis spp. (Seth-Smith et al., 2016), yet amplification with *Chlamydiae*-specific 16S rRNA gene primers also gave clear products which were cloned and sequenced.

**Figure 1:**
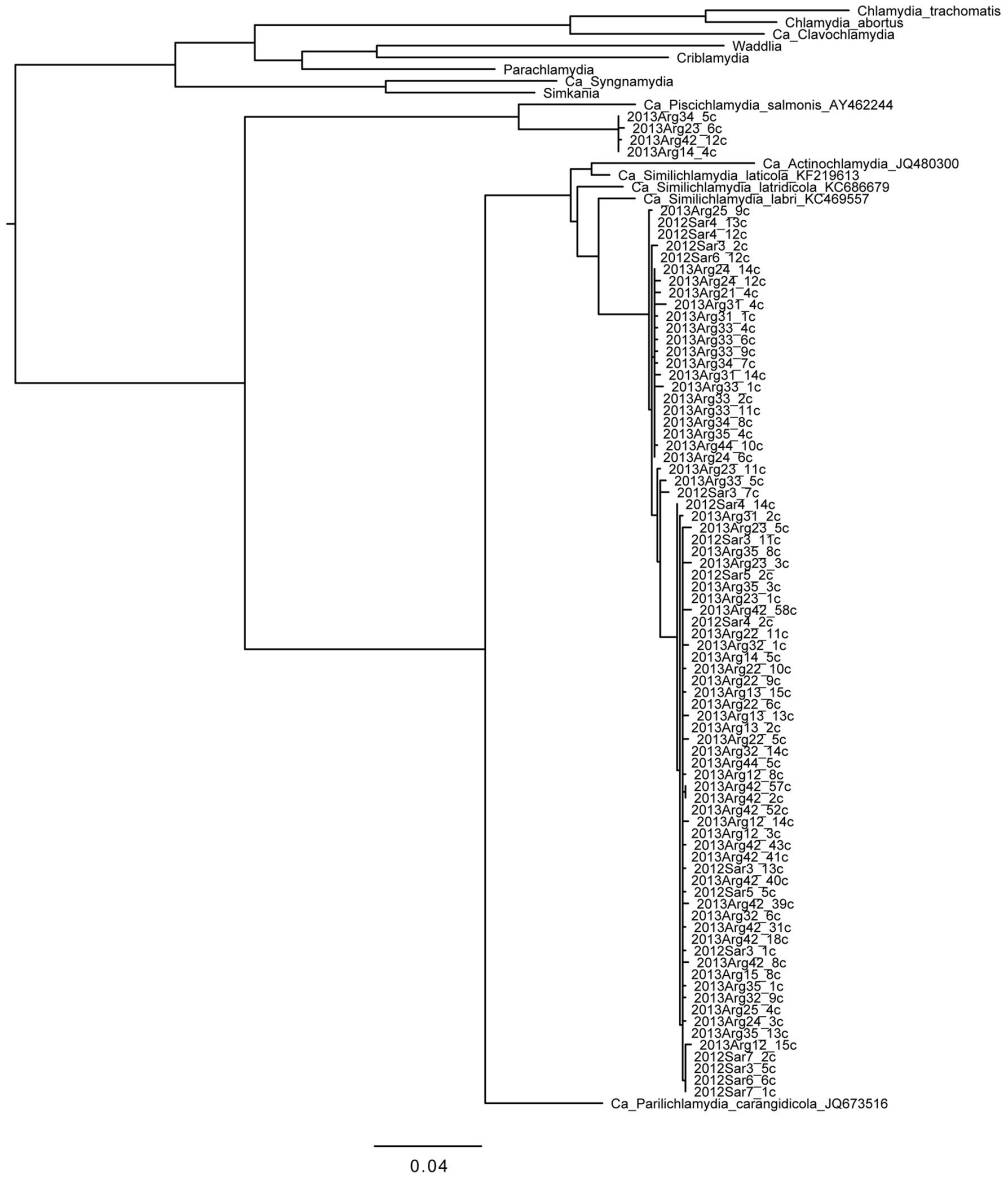
**Phylogenetic tree of chlamydial 16S rRNA gene sequences obtained from gilthead seabream gills**. Reference sequences from the phylum as a whole and from the deep-rooted “fish-*Chlamydia*” clade (provisional families *Ca*. Piscichlamydiae, Parilichlamydiae, Actinochlamydiae, and Similichlamydiae) are shown to put the sequences we obtained in this study in context. Clones from fish samples are named from the gill sample with the postscript “c” denoting amplification with *Chlamydiae*-specific primers. Four of the novel sequences from four fish from Argolida are more closely related to *Ca*. P. salmonis; 63 of the novel sequences from Saronikos and Argolida are more closely related to *Ca*. S. labri and *Ca*. S. latridicola. Sequences (~1,100 bp) were aligned and phylogenies generated within Seaview with 100 bootstraps. Branches separating the novel sequences from the reference sequences have bootstraps of 97% (*Piscichlamydiaceae* clade) and 100% (*Similichlamydiaceae* clade). Scale bar indicates number of substitutions per site.

Phylogenetic analysis of the 67 sequences from *Chlamydiae*-specific primers (Figure 1) showed 63 sequences, derived from all 21 fish studied from both Saronikos and Argolida, clustering very closely with each other and *Ca*. Similichlamydia. These share 97.2% nucleotide identity with a *Ca*. Similichlamydia labri sequence (accession number KC469554) over 1,075 bp from Norwegian ballan wrasse (Steigen et al., 2015) and 97% identity with *Ca*. Similichlamydia latridicola from striped trumpeter (Stride et al., 2013b). Thus, these may belong to a novel genus, but further full-length 16S rRNA gene sequencing would be required to confirm this (Yarza et al., 2014). There are two main clusters of sequences within this group: one of 19 sequences varying by 0–4 bp represented by 2013Arg33_2c, and the other of 41 sequences varying by 0–3 bp represented by 2013Arg23_1c.

Additionally, four sequences from four Argolida fish were analyzed and found to cluster with the *Ca*. Piscichlamydia and differ from each other by 0–2 bp. These share 94.1% identity over 1,091 bp with *Ca*. Piscichlamydia salmonis 16S rRNA gene sequence (accession number AY462244), indicating that they are likely to be distantly related members of this genus (Yarza et al., 2014). Thus, many novel strains of environmental *Chlamydia*, related to both *Ca*. Similichlamydia and *Ca*. Piscichlamydia, were found in the gills of this gilthead seabream cohort.

### Chlamydial load and morphological features of chlamydial cysts

**Figure 2:**
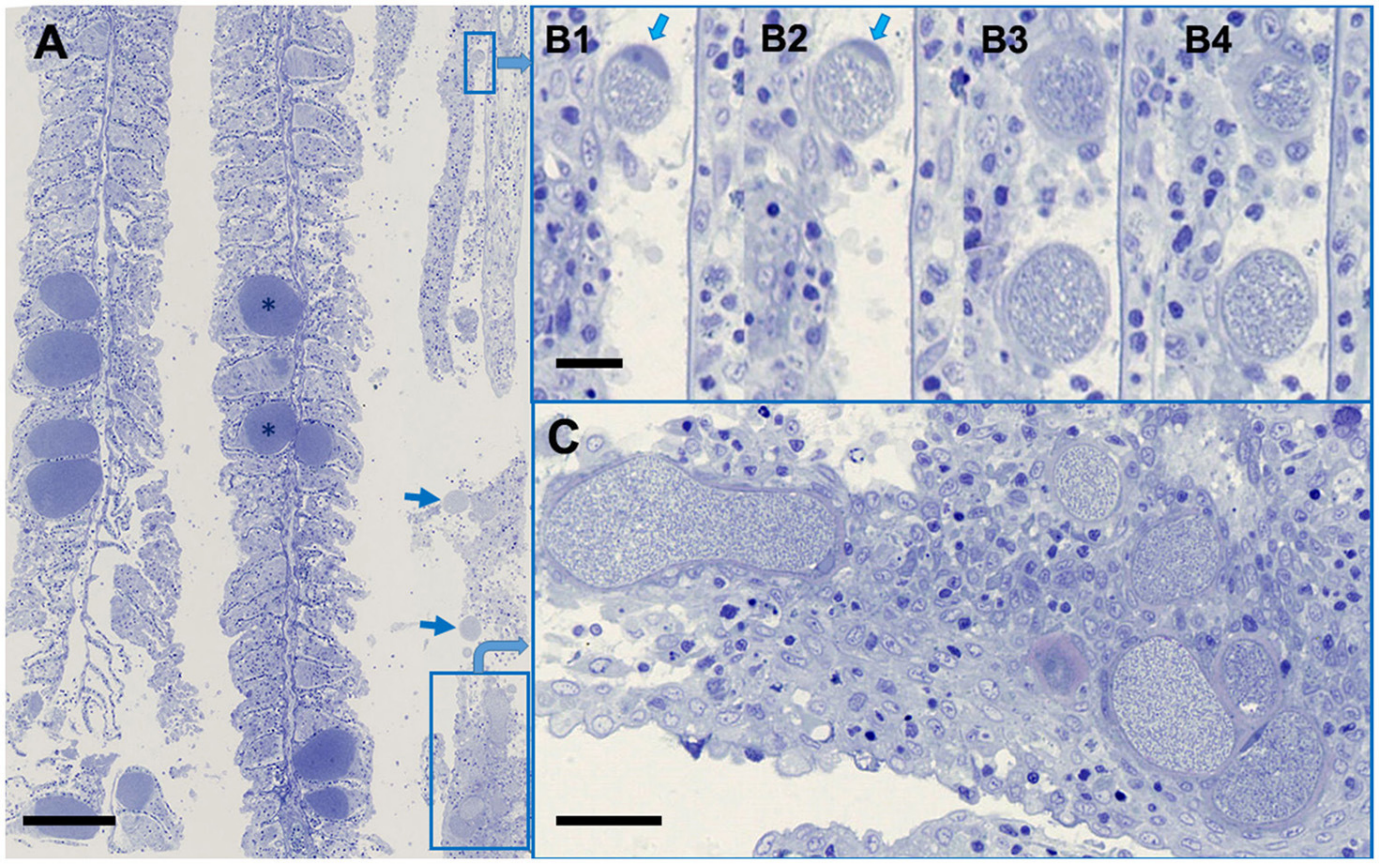
***Ca***. **Ichthyocystis sp. and *Ca*. Similichlamydia sp. cysts in adjacent gilthead seabream gill filaments. (A)** An overview, with the darkly stained *Ca*. Ichthyocystis sp. cysts (^*^) typically attached to the base of the primary lamella, and filling the space between two secondary lamellae, which become surrounded by proliferating epithelial cells, completely embedding the cyst. The *Ca*. Similichlamydia sp. cysts (boxed or with arrows) are lightly stained, often in clusters, and typically localized within the base of the primary lamella. **(B1–B4)** Serial 1 μm sections (although not sequential) of two small cysts with host cell nucleus (arrows) pressed between the inclusion and the cellular membrane. Regions shown were enlarged from region of small rectangular box, upper right of overview **(A)**. **(C)** Higher magnification of boxed region, lower right **(A)**, for better visualization of similichlamydial cysts, all with broad inclusion membranes. Semi-thin 1 μm sections were stained with toluidine blue and imaged using a Hamamatsu Nanozoomer slide scanner, equipped with a 25 x NA 0.75 objective lens, producing images with pixel sizes of 0.25 μm. Scale bars are 100 μm in **(A)**, 10 μm in **(B1–B4)**, and 25 μm in **(C)**.

Previous, quantitative analysis by qPCR comparing copy numbers of *Chlamydiae* and *Ca*. Ichthyocystis spp. showed that mixed infections were commonly observed, and the ratios of the infectious agents differed widely in individual fish, ranging from 1:5 to 1:3,000 (Seth-Smith et al., 2016, Supplementary Material). These results were reflected in semithin sections stained with toluidine blue (Figure 2), allowing a clear differentiation between the dominant darkly staining and densely packed cysts of *Ca*. Ichthyocystis sp. and the fewer, lightly staining, thick walled chlamydial cysts. In smallest chlamydial cysts, presumably reflecting earlier stages of development, the peri-nuclear location of the intracellular cyst is readily evident, pressed to the side of the cell, reminiscent of *Ca*. Piscichlamydia (Schmidt-Posthaus et al., 2012) or a typical chlamydial type inclusion (Taylor-Brown et al., 2015). This can be seen in serial sections of a small cyst shown in Figure 2, where the cell wall thickness becomes quite marked through the increasingly oblique sectioning angles, relative to the cyst. Even in larger, presumably later stage cysts, only a single nucleus deformed by the cyst inclusion is evident. The clustering of chlamydial cysts along one of the three gill arches in this section is helpful for illustrating the different cyst morphologies, although the proportion of chlamydial to *Ca*. Ichthyocystis sp. cysts is normally lower, consistent with the relative loads previously determined molecularly (Seth-Smith et al., 2016, Supplementary Material).

**Figure 3:**
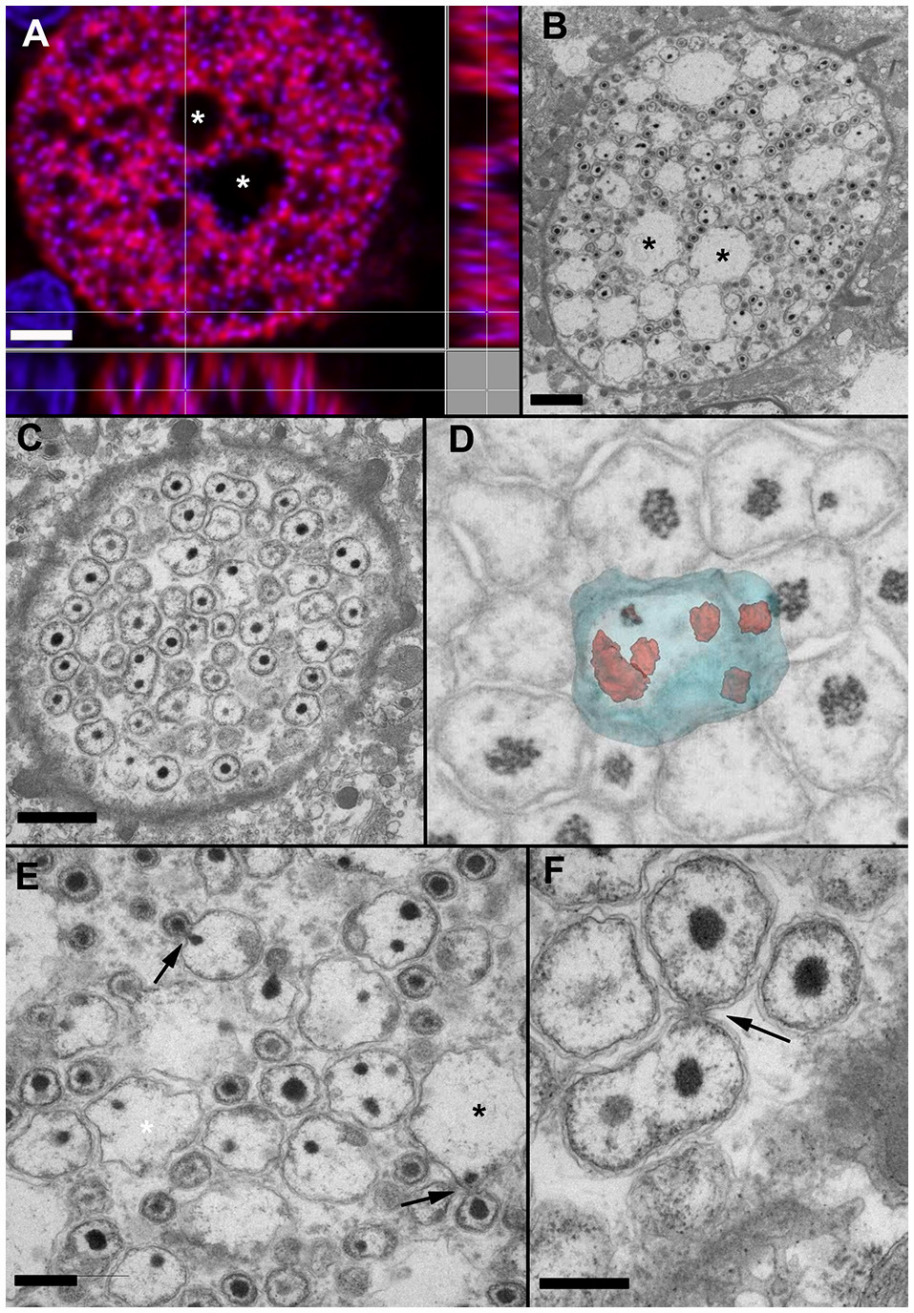
***In situ*** **hybridization (A)** and EM of early **(C,D)** and mid-stage **(A,B,E,F)** cysts. **(A)** Orthogonal projection of *Ca*. Similichlamydia labeled cyst from sample 2013Arg23, stained with Pisci0312-Cy3 in red and DAPI in blue and visualized with CLSM. **(B)** An equivalent cyst prepared for TEM, with similar vacuolar spaces (^*^) evident in both cysts and the condensed bacterial nucleoids of the IBs and EBs staining with DAPI in **(A)** and electron-dense in **(B)**. **(C)** At a putatively earlier stage, the cysts are filled with amorphous bacterial forms, which can contain multiple condensed nucleoids, shown here with TEM. **(D)** The forms in C can best be visualized using 3D imaging with FIB-SEM. Here is a snapshot from a 3D FIB-SEM stack (see Video S1) showing a segmented reconstruction of a large amorphous RB containing multiple condensed nucleoids. **(E)** A higher magnification image of **(B)** reveals what appears to be an asymmetrical budding of IBs, with individual bacteria first being enclosed by an outer membrane, prior to release from the RB. The large electron-lucent spaces may well represent remnant RBs, left over after the budding process is complete. **(F)** Asymmetrical budding of smaller RBs is also evident, as is a reticular network, often observed within the host cell and enclosing the inclusion, as can be seen in the bottom right hand corner of this image. Scale bars **(A,B)** (2 μm), **(C)** (1 μm), **(E,F)** (0.5 μm).

FISH with a probe recognizing both *Ca*. Similichlamydia and *Ca*. Piscichlamydia of this deep branching chlamydial clade, gave a positive signal only with the chlamydial cysts (Figure 3A) and did not react with the generally larger *Ca*. Ichthyocystis sp. cysts. Conversely, *Ca*. Ichthyocystis sp. specific probes only reacted with these larger cysts (Seth-Smith et al., 2016, Supplementary Figure 3), and did not detect the chlamydial cysts.

Using the semithin sections for orientation, ultrathin sections were prepared and similichlamydial cysts were selected for TEM, whereby an attempt was made to cover as many stages of the putative developmental cycle as possible (Figure 3). The broad cell walls of smaller early stage cysts, detected in histology, revealed itself to be an intricate tubular or vesicular network, often enmeshing mitochondria as well as darkly staining deposits, possibly lipid containing, and intimately connecting with the chlamydial inclusion membrane (see also Video S1). In contrast to clavichlamydial cysts (Steinum et al., 2010; Schmidt-Posthaus et al., 2012), we detected no projections penetrating the inclusion membrane and associated with or attached to dividing (RB-like) bacteria. Dividing bacteria were detected throughout the putative early stage inclusions (Figures 3C,D,F, Video S2), indicative that nutrients from the host and within the inclusions are either freely solubilized in the inclusion liquor or their transport utilizes vesicular-based mechanisms. Rather than a replication initiated by simple division of RBs, once they reach a critical mass, the RBs appear quite amorphous in size and form, with up to eight nucleoids detected within a single RB (Figure 3D and Video S2). Here the use of FIB-SEM was invaluable in unequivocally visualizing the multinucleoid nature of these amorphous RBs. New bacteria appear to arise by budding (Figures 3B,C,E,F), which always occurs at one position from a given parent cell, indicative of a polar process. This is especially marked in the budding bodies shown in Figure 3E, where condensed nucleoids can be seen on both sides of the neck connecting the budding cells. The loose, wavy bacterial membranes and the many filamentous particles within the bacterial bodies are likely to be artifacts of the fixation and embedding process.

**Figure 4:**
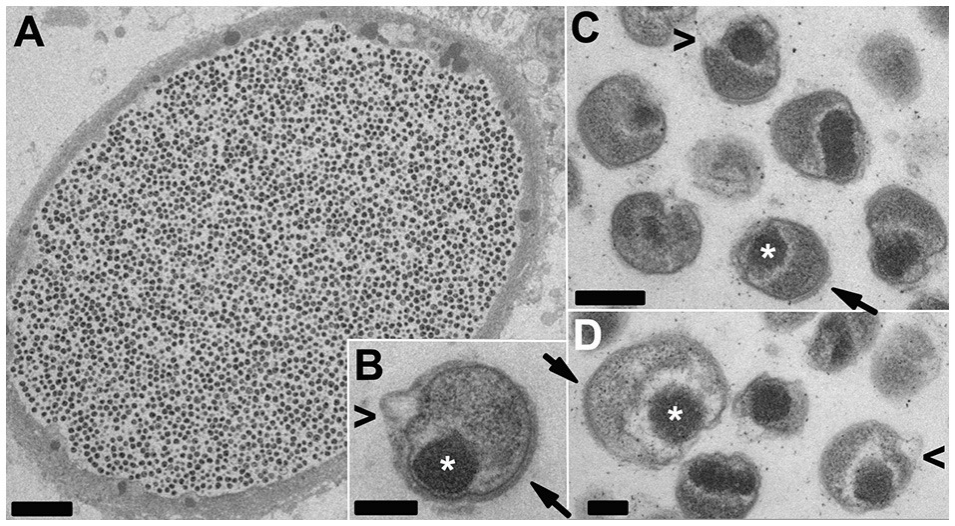
**TEM of putative late-stage *Ca*. Similichlamydia cysts containing uniform populations of EB-like forms**. An overview of an entire cyst **(A)** together with a selection of higher magnification images of individual EBs **(B–D)** showing tightly condensed chromosomes (^*^) associated with the bacterial membrane in a polar location and adjacent or opposite, bacterial membrane spanning actinae (filaments, arrows) projecting away from the outer bacterial membrane. The EBs appear to reduce in size and become more compact by the pinching off of outer membrane vesicles (arrowheads < in **B–D**), although this may also be a shrinking artifact of the chemical fixation methods used. Scale bars **(A)** (5 μm), **(B,C)** (0.2 μm), **(D)** (0.1 μm).

Putative late stage inclusions (Figure 4) contain uniform populations of EB-like forms, with features similar to those described for the closely related *Ca*. Actinochlamydia (Steigen et al., 2013). A condensed nucleoid is typically localized in a polar fashion adjacent to the bacterial membrane with an array of filaments (actinae, Steigen et al., 2013) penetrating the membrane on an adjacent or opposite side, indicative of a polarized bacterial cell with clearly defined functional regions.

## Discussion

Investigation of the deepest rooting *Ca*. Piscichlamydia clade within the phylum *Chlamydiae* is attractive for exploring the origins of this large phylum (Lagkouvardos et al., 2013) and highly successful group of obligate intracellular bacteria. In particular, morphological and molecular features shared with the sister phyla of the *Planctomycetes* and *Verrumicrobiae* of the PVC superphylum may aid us in understanding how these bacteria evolved. The *Chlamydiae* are all thought to progress through a biphasic developmental cycle, initiated by infection of a susceptible host epithelial cell with the infectious particles or EBs, which are taken up and enclosed in a membrane bound inclusion, whereupon larger replicative forms of dividing bacteria (RBs) multiply to fill the inclusion before condensing to form EBs, which are released from the cell to initiate a fresh infectious cycle. Both the *Chlamydiae* and members of the *Planctomycetes* lack the tubulin homolog FtsZ which is involved in forming the septum regulating the binary fision of most bacterial cells, raising the question as to how this process is regulated. In FtsZ-less members of the *Planctomycetes* this occurs by a polar budding of bodies from a larger intermediate form (Fuerst, 1995; Lee et al., 2009; Santarella-Mellwig et al., 2013). Elements of these processes may be present in this deep rooted *Ca*. Piscichlamydia/Similichlamydia clade.

Several features stand out. During the replicative stage, the RBs are amorphous in form and size and can contain multiple nucleoids, with a new bacterium being generated via a single polar budding process. We only observed the budding of a bacterium with a single nucleoid and did not find evidence for cleavage of a multi-nucleoid containing RB into two RBs, each still with multiple nucleoids. This would indicate that the budding process is a tightly regulated one. We have previously described what appeared to be budding RBs in TEM images of *Ca*. Piscichlamydia from epitheliocystis lesions in brown trout (*Salmo trutta*, Schmidt-Posthaus et al., 2012), indicating that this mechanism may be common for the entire deep rooted clade comprising the four putative families (*Ca*. Piscichlamydia, Similichlamydia, Parilichlamydia, Actinochlamydia), all members of which have only been found as intracellular bacterial pathogens of marine or fresh water vertebrates, indicative of their early evolutionary origins and specialization for an aqueous environment (Draghi et al., 2004; Schmidt-Posthaus et al., 2012; Steigen et al., 2013, 2015; Stride et al., 2013a,b,c). Curiously, the large RBs with multiple nucleoids which we find commonly here (Figures 3C,D) are more reminiscent of the aberrant bodies generated by antibiotic treatment or nutrient restriction in the *Chlamydiaceae* (Polkinghorne et al., 2006). *Ca*. Similichlamydia RBs are also distributed throughout the inclusion and do not appear to be preferentially aligned along the inclusion membrane with attachment through filaments or projections, as has been observed for the *Chlamydiaceae* or for their immediate marine relatives, the *Clavichlamydiaceae* (Schmidt-Posthaus et al., 2012).

Another feature is the small (0.3–0.5 μm) densely packed particles (Figure 4), resembling the infectious particles or EBs of the *Chlamydiaceae*, (Taylor-Brown et al., 2015) albeit with a polar form and clusters of membrane spanning filaments or actinae, first described in the *Ca*. Actinochlamydia (Steigen et al., 2013), which could temptingly be postulated to influence host recognition and uptake. A major caveat, indeed a caveat which applies to the majority of the EM studies with *Chlamydia*, is that the use of chemical fixation combined with classical dehydration procedures prior to embedding can lead to a range of artifacts and poor membrane preservation. However, due to the remoteness of the fish farms, we had no possibility of applying advanced cryo-EM techniques as used to excellent effect by others (i.e., Santarella-Mellwig et al., 2013) to the samples we collected, and which would have aided us greatly in the interpretation of morphological features. This is a possibility for the future, but would require setting up of aquarium facilities close to the EM facilities and stocking these with infected fish, not a trivial undertaking. Conversely, should we one day finally succeed in isolating and cultivating these bacteria, this will be one of the experimental priorities.

It was long thought that the *Chlamydiaceae* RBs undergo binary fission, as most other bacteria, prior to transformation into the infectious particles or EBs. This may not be the case. As we were preparing this manuscript, an elegant study was forthcoming, indicating that at least with *Chlamydia trachomatis*, EBs are generated by polar budding from RBs, possibly also containing multiple chromosomes (Abdelrahman et al., 2016), reminiscent of the structures we present here for *Ca*. Similichlamydia (Figure 3). This study relies strongly on excellent high resolution immuno-light microscopy imaging, and identifies a number of chlamydial proteins which may have a role in regulating this process. It would be intriguing to investigate whether homologs of these proteins are encoded by the similichlamydial genome, and if so, how these proteins might be regulated.

Indeed, a concerted effort to analyse the genomes of this deep-rooted chlamydial clade would provide an invaluable insight into the essence of what makes a *Chlamydia* and how the members of this phylum share their origins within the PVC superphylum. Detailed morphological studies are critical to these efforts (Draghi et al., 2004; Schmidt-Posthaus et al., 2012; Steigen et al., 2013, 2015), to complement genomic analyses, which have now come within reach (Katharios et al., 2015; Qi et al., 2016; Seth-Smith et al., 2016).

## Author contributions

Conceived and designed the experiments: AF, HSS, JM, MR, ND, PK, and LV. Performed the experiments: AF, HSS, JM, LN, MS, MR, ND, PK, and LV. Analyzed the data: AF, HSS, JM, LN, MR, MS, PK, and LV. Contributed reagents/materials/analysis tools: HSS, JM, MS, ND, and PK. Contributed to writing the manuscript: HSS, JM, MR, PK, and LV.

## Funding

This work was supported by the European Union through Marie Curie Intra-European Fellowship grant number 332058 to HSS and an FP7 Aquaexcel-TNA project 01-05-15-0004-B to LV and PK. AF was partly supported by SNF project 310030_138533 to LV. The funders had no role in study design, data collection and analysis, decision to publish or preparation of the manuscript.

### Conflict of interest statement

The authors declare that the research was conducted in the absence of any commercial or financial relationships that could be construed as a potential conflict of interest.

## Supplementary material

Video S1 **Video of sequential Z-slices of a portion of a cyst taken with FIB-SEM showing the inclusion membrane (putative outermembrane)**. The broad cell wall of smaller early stage cysts, detected in histology, revealed itself to be an intricate tubular, or vesicular network along the outer side of the inclusion membarne, often enmeshing mitochondria as well as darkly staining deposits, possibly lipid containing, and intimately connecting with the chlamydial inclusion membrane. The amorphous nature of the RBs and the variable number of electron dense nucleoids can readily be seen.

Video S2 **Video of a portion of a cyst taken with FIB-SEM showing a segmented reconstruction of a large amorphous RB containing multiple condensed chromosomes**. In this orthogonal projection, the electron-dense material, bacterial chromatin, or nucleoids, is rendered in red and the outer surface of the bacteria in blue. The number of nucleoids per bacteria is variable, indicative that replicating bacteria at various stages of growth are present. The voxel size in both videos is 5 × 5 × 20 (XYZ).

## Abbreviations

FIB-SEM: focused ion beam-scanning electron microscopy
TEM: transmission electron microscopy
CLSM: confocal laser scanning microscopy
EB: elementary body
RB: reticular body
IB: intermediate body
FISH: fluorescent *in situ* hybridisation.
